# Social-demographic shift in drug users at the first-ever- methadone maintenance treatment in Wuhan, China

**DOI:** 10.1038/s41598-017-11888-5

**Published:** 2017-09-13

**Authors:** Cong Liu, Pu-lin Liu, Quan-lin Dong, Li Luo, Jun Xu, Wang Zhou, Xia Wang

**Affiliations:** 1 Wuhan centers for disease prevention and control, Hubei province, China; 2 Department of Epidemiology and Biostatistics, School of Public Health, Tongji Medical College,, Huazhong University of Science and Technology, Hubei province, China

## Abstract

The methadone maintenance treatment (MMT) has been initiated in Wuhan, China since early 2006. To understand the social-demographic, behavioral, and infectious diseases characteristics of drug users enrolled in their first-ever-MMT between 2006 and 2015, a retrospective observational study was implemented to also provide evidence for health policy-decisions to reduce harm and control disease. Pearson chi-square tests and t-tests were used to assess significant differences between two 5-year periods, 2006–2010 and 2011–2015. We observed increases in the mean age (38.65 vs. 42.43 years, P < 0.001), mean age of initial opioid drug use (28.18 vs. 31.07 years, P < 0.001), employment (11.9% vs. 30.7%, P < 0.001), married/co-habiting (42.4% vs. 47.8%, P < 0.001), and declines in higher education level (93.6% vs. 84.8%, P < 0.001), injection (82.3% vs. 75.1%, P < 0.001), syringe sharing (27.7% vs. 9.9%, P < 0.001), HCV infection rates (72.9% vs. 70.5%, P = 0.017). The number of drug users enrolling each year reduced following a continuous rapid growth in the first 3 years. The findings imply for adjusting in treatment services and allocation of resources to respond to emerging trends. In addition, the data will also be helpful for identifying needs and getting a baseline insight of the social-demographic and behavioral characteristics of the opioid abusers in the area.

## Introduction

Drug abuse is a serious public health problem that plagues the entire world^1^. Currently, as well as in China’s past, drug abuse has significantly impacted public health, economic development, and social harmony^2–4^. Since 1980, the drug abuse epidemic reemerged with China’s economic and social liberalization, and has continued to develop^5^. By the end of 2012, there were approximately 1.27 million opioid abusers. This number accounted for 60.6% of the 2.09 million known drug users registered by judicial authority^6^. Because the typical route of drug administration in these users was by intravenous injection (50–70%)^7, 8^, and many of these drug users were also known to engage in high-risk sexual behaviors^9, 10^, both intravenous injection and high-risk sexual behavior were associated with HIV transmission^11–13^. To reduce high risk behavior, and thereby HIV infection, among these drug users, the Chinese public health decision-makers implemented methadone maintenance treatment (MMT). This has been in place since 2004, and is the only community treatment modality in China.

MMT has proved to be successful in the treatment of those with an opioid dependence, resulting in a reduction in opioid abuse^14–18^. MMT was also demonstrated to be effective in reducing high-risk behaviors associated with HIV transmission, thus preventing the spread of HIV among drug addicts^19–22^. In addition, MMT has been shown to increase the initiation and adherence of patients to antiretroviral therapy (ART)^23–27^, decrease the risk of ART discontinuation^28, 29^, and improve overall treatment outcomes^30–33^ in HIV-positive opioid addicts. Numerous studies have shown that demographics play an influential role in determining whether opioid drug users benefit from the MMT program. While females were found to be more likely to carry out illicit opioid use during MMT treatment^34^, males were found to be likely to engage in high-risk sexual behaviors^35^. Another study determined that while there were no significant differences in risk behaviors between genders, the odds of HIV infection among male opioid drug users were significantly higher compared to females in high transmission areas. Interestingly, the opposite was found to be true in low transmission areas^36^. Meta-analysis studies also determined there was no significant difference in HCV infection between male and female IDUs^37^. Age was found to have a paradoxical association with concurrent opioid drug use^34, 38^. However, younger subjects (<30 years) were associated with a higher risk of HCV infection^36^ and a higher drop-out rate^39, 40^. While the age of initial opioid drug use (<25 years)^41^ and high education level^39, 42^ were found to be positively associated with a higher probability of continuous treatment retention, a younger age of initial opioid drug use (<18 years) was found to be at a higher risk of having a physical or psychiatric comorbid disorder^43^. Unemployment^44, 45^, not being married^45^, were independently associated with premature MMT discharge at 6 months, while young males (<35 years) were associated with a premature MMT discharge at 12 months^45^. In addition, a stable income was found to be a protective factor associated with MMT retention^40^. In summary, the demographics of drug users receiving MMT provide valuable information to psychiatrists for providing targeted drug abuse treatment plans and psychotherapy. These data are also beneficial for the analysis of the efficacy of MMT programs.

Wuhan is the capital of Hubei Province and is the most populous city in Central China. This city serves as the political, economic, and cultural center of Hubei Province. The city of Wuhan has a population of approximately 10,607,700 people, with a GDP of 119,126.1 million yuan (RMB). The number of drug users in 2015 was estimated to be approximately 20,000 by the Workbook model. This model focuses on identifying populations that demonstrate behaviors that place them at high risk of HIV infection, or who are exposed to HIV through the risk behavior of their sexual partners^46, 47^. According to national scale-up plans, MMT programs were initiated in Wuhan city in 2006. To date, a total of 17 MMT clinics have been established, with approximately 4000 patients currently undergoing treatment.

A nationwide study demonstrated that the profile of clients enrolling in MMT in China was continually changing. This included the mean age of participants, increased numbers of participants who were married or living together, and decreased numbers of participants who were unemployed or between jobs, or had an accepted higher education, administered drugs via injection, shared needles, were HIV positive, and HCV infection^48^. However, there has been no study carried out towards gaining an understanding of the changes in socio-demographic and serobehavioral characteristics of opioid drug users at the first-ever-MMT treatment. We hypothesize that similar changes existed in opioid drug users at the first-ever-MMT treatment in Wuhan over the past 10 years. Thus, adjustments in treatment services and the allocation of resources would be required in order to respond to emerging trends.

Here, we describe the demographic characteristics, behavior, and serological status of opioid drug users at the first-ever-MMT. Additionally, we attempt to understand the changing trends that occurred between 2006 and 2015. In the meantime, we provide evidence for local health sectors to enable the tailoring of policies and strategies to reduce high-risk behaviors and related HIV/HCV infection as a result of drug abuse.

## Methods

### Data

The data used in this study was obtained through electronic medical records sourced from the national MMT data management system. Data collection was carried out from January 1, 2006 to December 31, 2015. Each case in the database was counted once, and the analysis was “person-based”. Demographic and behavior characteristics data were collected through face-to-face interviews carried out by doctors. Blood samples were obtained to test for HIV, hepatitis C virus (HCV), and syphilis at the first treatment entry of participants.

To determine the HIV status of participants, an algorithm was used which combined results from an initial test based on the enzyme-linked immunosorbent assay (ELISA) method and two tests to detect HIV antibodies. If positive results were obtained, a second test was carried out using the Western blot method. HCV and syphilis were detected using antibody-based assays.

All doctors of MMT clinics had been trained for interview by Wuhan centers for disease prevention and control. The data collection of electronic medical records was carried out in accordance with The Guidelines of AIDS Prevention among Drug Users at MMT Clinics. All experimental protocols were approved by the Chinese Center for Disease Control and Prevention.

### Participants

All participants voluntarily applied for MMT with informed consent, and were required to fulfill the following 5 criteria: (1) participant must be in their first-ever-MMT; (2) participant must be 18 years old or above; (3) participant must have a history of opioid use, with the initial use of the primary substance being opioid drugs; (4) participant must have complete mental capacity; (5) participant must provide an opioid positive urine specimen prior to enrollment. Drug users who did not meet the above criteria were excluded from this study.

### Statistical analysis

To compare the social-demographic characteristics, behavioral characteristics, HIV status, HCV status, and syphilis status between different time periods, all participants were grouped according to their first-ever-MMT date. In view of the fact that there was no MMT clinic established in Wuhan city after 2010, we split the 10-year period into two phases. The first phase, phase I (2006–2010) was considered to be the development phase of MMT initiation, and the second phase, phase II (2011–2015) was considered the stable implement phase of the MMT program. We sought to analyze differences between these two phases.

The data obtained from the study was abstracted from the national MMT data management system, and the database was managed by Microsoft Excel 10.0. Statistical analyses were performed using SPSS 19.0 for Windows. We used descriptive statistics to summarize the demographic and behavioral characteristics, and the HIV, HCV, and syphilis infection status of participants. We calculated the Pearson correlation coefficient to determine the relationship between the mean age at enrollment and the mean age of initial opioid drug use. Continuous variables were expressed using the mean with the standard deviation and categorical variables using percentage. To test for significant differences, we used the Pearson chi-square test for categorical variables and the t-test for continuous variables. All P-values were two-sided and P < 0.05 was considered to be significant.

### Data Availability

The data that support the findings of this study are available from national MMT data management system but restrictions apply to the availability of these data, which were used under license for the current study, and so are not publicly available. Data are however available from the authors upon reasonable request and with permission of Chinese Center for Disease Control and Prevention.

## Results

**Figure 1:**
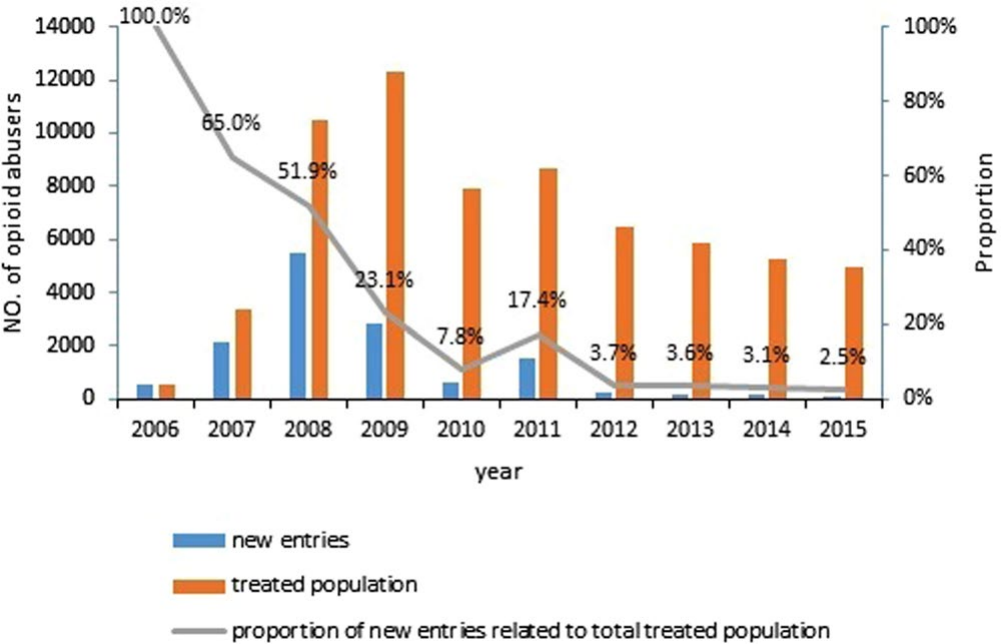
The number of drug users enrolled in the MMT program.

According to the criteria of participants, there was a total of 13,884 drug users that had their first-ever-MMT in Wuhan city between 2006 and 2015. During this period, the number of drug users who accepted their first-ever-MMT increased in the first 3 years, but decreased in the last 7 years. The proportion of new entries relative to the total treated population was found to have decreased dramatically from 2006 to 2010, increased slightly in 2011, followed by another decreased and bottoming out after 2011 (Fig. 1).

The mean age of drug users at entry in the program was 39.26 years (SD = 7.570), with a mean age of initial opioid drug use of 28.64 years (SD = 7.915). Among all drug users, the majority were male (73.8%), ethnic Han (99.0%), unemployed (85.0%), and received junior middle school or higher education (92.2%). Less than half of the drug users were married or lived in a cohabitation setting (43.3%). The majority were reported to have injected drugs (81.1%) in the last six months (74.4%). A total of 24.4% of participants reported having shared needles in the three months prior to enrollment. Biological test results showed that 0.4% of the enrolled drug users were HIV-positive, 72.5% were HCV-positive, and 3.1% were syphilis-positive at the time of enrollment (Table 1).

**Table 1:** The characteristics of drug users at the first-ever-MMT in Wuhan city, China for the 10-year period 2006–2015, and separately for the phase I (2006–2010) and phase II (2011–2015).

characteristics		Total N = 13884	2006–2010 (phase I) N = 11636	2011–2015 (phase II) N = 2248	P
age at entry*					
	mean years	39.26	38.65	42.43	<0.001
	sd	7.57	7.312	8.08	
age of initial opioid drug use*					
	mean years	28.64	28.18	31.07	<0.001
	sd	7.915	7.613	8.94	
gender					
	male	10252(73.8%)	8559(73.6%)	1693(75.3%)	0.083
	female	3632(26.2%)	3077(26.4%)	555(24.7%)	
ethnic group					
	Han	13752(99.0%)	11535(99.1%)	2217(98.6%)	0.022
	non-Han	132(1.0%)	101(0.9%)	31(1.4%)	
marital status					
	Married or in cohabitation	6012(43.3%)	4938(42.4%)	1074(47.8%)	<0.001
	other	7872(56.7%)	6698(57.6%)	1174(52.2%)	
education level					
	junior middle school or above	12802(92.2%)	10895(93.6%)	1907(84.8%)	<0.001
	under junior middle school	1082(7.8%)	741(6.4%)	341(15.2%)	
employed					
	no	11805(85.0%)	10247(88.1%)	1558(69.3%)	<0.001
	yes	2079(15.0%)	1389(11.9%)	690(30.7%)	
ever injected drugs					
	no	2621(18.9%)	2061(17.7%)	560(24.9%)	<0.001
	yes	11263(81.1%)	9575(82.3%)	1688(75.1%)	
drug use in last 6 months					
	only injected	10327(74.4%)	9081(78.0%)	1246(55.4%)	<0.001
	non-only injected	3557(25.6%)	2555(22.0%)	1002(44.6%)	
share needles in last 3 months					
	no	1186(75.6%)	922(72.3%)	264(90.1%)	<0.001
	yes	382(24.4%)	353(27.7%)	29(9.9%)	
HIV status					
	negative	13835(99.6%)	11592(99.6%)	2243(99.8%)	0.254
	positive	49(0.4%)	44(0.4%)	5(0.2%)	
HCV status					
	negative	3816(27.5%)	3152(27.1%)	664(29.5%)	0.017
	positive	10068(72.5%)	8484(72.9%)	1584(70.5%)	
Syphilis status					
	negative	13438(96.8%)	11247(96.7%)	2191(97.5%)	0.047
	positive	446(3.2%)	389(3.3%)	57(2.5%)	

**Figure 2:**
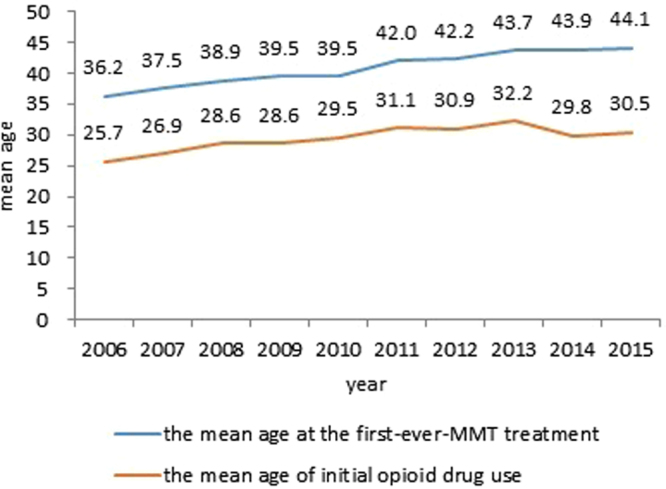
The mean age of drug users enrolled in the MMT program.

The mean age of initial opioid drug use or age at entry to the program was found to increase with each year (Fig. 2). Comparing the phase I group (2006–2010) with the phase II group (2011–2015), we found that the mean age of drug users at enrollment increased by 3.78 years (38.65 vs. 42.43, P < 0.001) and the age of initial opioid drug use increased by 2.90 years (31.07 vs. 28.18, P < 0.001). In addition, we calculated the Pearson correlation coefficient to be 0.789 (P < 0.001) between the mean age of drug users at the time of enrollment and the mean age of initial opioid drug use between 2006 and 2015.

While there were no significant changes based on gender (P = 0.083) between the two phase groups, the number of ethnic Han participants was found to decrease from 99.1% to 98.6% (P = 0.022), unemployment was found to decrease from 88.1% to 69.3% (P < 0.001), the number married or cohabiting was found to increase from 42.4% to 47.8% (P < 0.001), and the number with junior middle school education or higher was found to decrease from 93.6% to 84.8% (P < 0.001). In addition, the number of drug users who had ever injected drugs decreased from 82.3% to 75.1% (P < 0.001) and intravenous (IV) drug use in the six months prior to enrollment decreased from 78.0% to 55.4% (P < 0.001). The number of participants who had shared needles in the three months prior to enrollment also decreased from 27.7% to 9.9% (P < 0.001) (Table 1). The HCV-positive rate decreased from 72.9% to 70.5% (P = 0.017), the syphilis-positive rate decreased from 3.3% to 2.5% (P = 0.047), and the HIV-positive rate decreased slightly (by 0.2%), but showed no significant difference (P = 0.254) (Table 1).

## Discussion

The findings of our study suggest that there was a significant change in the social-demographic and behavioral characteristics of drug users upon enrollment in the MMT program in Wuhan over time. We found that the number of participants enrolling in the program grew rapidly within the first few years, followed by a sharp reduction. The mean age of both initial opioid drug use and age at enrollment were both found to increase. In addition, we found that while the percentage of those employed, married or in cohabitation situations increased, the percentage of those with junior middle school or higher education declined. High risk–related injection behavior was found to significantly decrease, corresponding with a slight decrease in HCV and syphilis rates. While the number of HIV-positive individuals also decreased, the difference observed did not reach the pre-defined levels of significance.

The number of drug users enrolled in the first-ever-MMT were found to decrease year by year following a continuous rapid growth in the first three years. The proportion of new entries relative to the total treated population was found to decline. This was attributed to a number of factors, with the most important factors being continuous efforts to address opioid addiction^49, 50^ and increases in the use of novel psychoactive substances (NPS)^5, 6^. Because of the dangers associated with opioid use for the nation, as demonstrated in history by the Opium War^49^, the Chinese government at all levels has always ensured proper health education and intervention of opioid addiction was available to the public^5, 49, 51^. Due to the risk of HIV transmission with IV drug use (such as heroin)^3, 49, 51^, similar to other pilot cities, the local authority of Wuhan initiated the MMT program for the treatment of opioid addicts in 2006, and promoted peer education and 100% MMT program among drug users within the first 5 years (2006–2010). Previously, it was a widely held belief that NPSs were less addictive and harmful compared to opioids^6^, with a potential shift towards NPSs from opioids^5^. Increases in the use of NPS^5, 6^ garnered public attention, leading the health sector, police department, and education system to collaborate in order to strengthen intervention policies and decrease NPS use, particularly among the younger population. Fluctuations in the number of opioid abusers entering their first-ever-MMT over time could be related to changes in MMT clinics, as well as the estimated number of opioid abusers in Wuhan city. The number of MMT clinics increased sharply from 0 to 20 in 2006–2008, levelled off until 2010, then increased and levelled off at 22 in 2011–2014. This number then decreased to 21 in 2015. In addition, the estimated number of opiate abusers sharply increased in 2006, summited in 2009, sharply decreased in 2010, and gradually decreased following 2010. Thus, the sharp increases in the first-ever-MMT entries were associated with the opening of new MMT clinics in 2006–2008. Similarly, the estimated number sharply decreased when the number of MMT clinics remained steady, and sharp decreases in the number of first-MMT entries may be normal after 2008. We suggest that the health care system should take further action to counter the increasing variation in drug abuse. Specifically, this would include a greater input in resources to train the MMT staff primarily in dealing with the needs associated with the use of NPS, the establishment of a referral system between MMT clinics and psychiatric hospitals, and an expanded capacity for NPS treatment in psychiatric hospitals.

The mean age of drug users between the phase groups was found to increase corresponding to the age of initial opioid drug use. This implied a link that is supported by existing literature^52^. This change in age could be due to younger people choosing to use NPSs over opioids^5, 6^ as well as the overall decrease in the use of opioids by younger people^5^. There were significant changes identified in employment, education level, and marital status. Decreased unemployment levels and increased numbers of those married or cohabiting participants demonstrate that a greater number of opioid drug users who entered into the MMT program are already a part of society. This suggests that the public has a greater tolerance to drug users who are willing to change^53, 54^. The decrease in the ratio of drug users who received a junior middle school education or higher indirectly suggests that those who received a higher-level of education are less likely to use opioids^55^. This could be attributed to in-school health education and drug abuse awareness^56^.

In regard to high risk behaviors, the proportion of IV drug use was found to have changed dramatically. The proportion of participants that self-reported ever having injected drugs, injected drugs in last six months, or had shared needles in the three months prior to enrollment was found to decrease significantly. Our findings of a reduction in high-risk behaviors were consistent with previous studies that have shown that continual long-term behavioral intervention^57–59^, educational intervention^60^ and peer education^61^ can result in reduced IV drug use and reduced sharing of injection equipment^62^.

**Figure 3:**
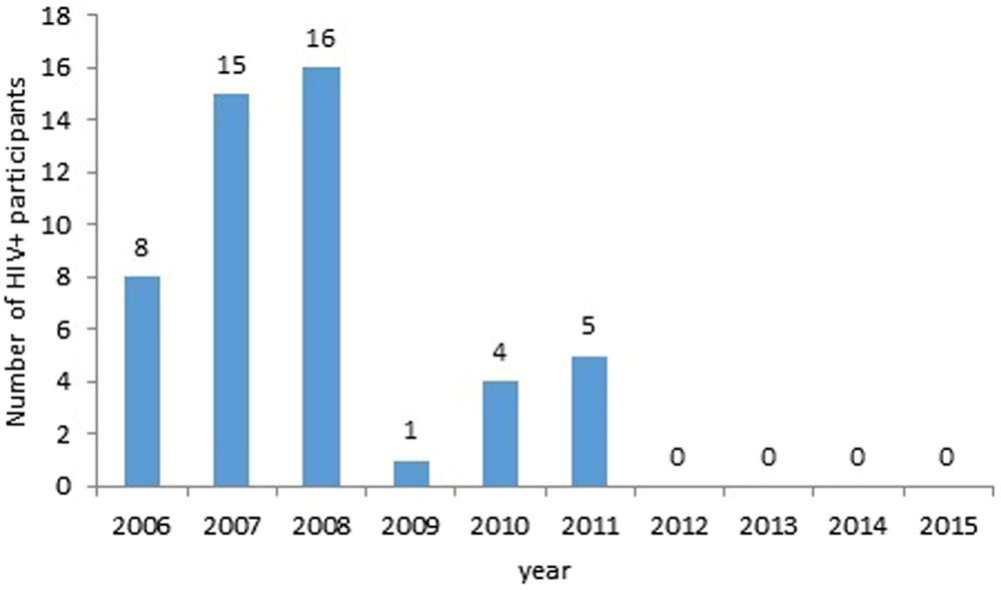
The number of HIV-positive among drug users at the first-ever-MMT entry.

Analysis from our study demonstrated that the rate of HCV-positive subjects was significantly decreased between the two-phase groups studied. Our findings that link high-risk injection practices with the HCV-positive rate seemingly further indicate that interventions can successfully reduce high-risk injection behavior, but have little effect in the prevention of HCV transmission among people who inject drugs^62, 63^. Further studies are needed to understand the impact of intervention programs on HCV infection rates among drug users. We also found a small decrease in the number of Syphilis-positive participants. We also found that the rate of Syphilis-positive retained a relatively low level in past 10 years, consistent with data from sentinel surveillance in Wuhan. In this study, we observed no significant difference in the HIV-positive rate decrease between the phase I group and phase II group. However, the data demonstrated that the number of HIV infections in drug users was reduced over the past ten years, with no HIV positive participants entering the Wuhan city MMT program between 2012 and 2015 (Fig. 3). The reasoning behind this observation is complex, and further studies will be required to fully understand this.

Our study also demonstrated a significant change in ethnicity, but not gender, between the two groups studied. The data shows that the male and Han-ethnic populations remain the largest group of drug users to receive MMT. However, the observed reduction in Han-ethnic participants requires further investigation to fully understand.

As is the case in all observational studies, the study described here possesses certain potential limitations. First, our research study was a cross-sectional study. Thus, the sample was not random, and caution should be taken when extrapolating our conclusions beyond the study group. Second, certain variables were self-reported by participants and are socially sensitive subjects. This could have resulted in a bias towards the more socially desirable options^64^. Third, the data collection system involving MMT did not collect information regarding use of secondary substances. Thus, we are unable to analyze the effect things such as polydrug use (including NPS) on changes of MMT entries. Despite the above limitations, the data collected from this study suggests that the social-demographics and behaviors of drug users at the first-ever-MMT have changed over the course of the past decade. The data collected from this study can also be used to monitor the basic status of drug users in a community, and could help to establish a baseline for further studies aimed to assess the MMT program. In addition, this data can be used to guide local planning efforts for controlling the spread of HIV and HCV among drug users in Wuhan.

## Conclusion

Over the course of the past decade, the social-demographic and behavioral characteristics of drug users that have enrolled in their first-ever-MMT treatment in Wuhan, China have changed. The findings from this study have significant implications for health sectors aiming to optimize the allocation of resources and improve the tailoring of treatment to specific patient groups. This would include the input of more resources towards the training of MMT staff to deal with NPS abuse, the expansion of the capacity for NPS treatment in psychiatric hospitals, and the establishment of a referral system between MMT clinics and psychiatric hospitals. The data collected from this study could also be used to establish a baseline for the support of further studies aimed to assess the MMT program.
